# Development of a bio-optical model for the Barents Sea to quantitatively link glider and satellite observations

**DOI:** 10.1098/rsta.2019.0367

**Published:** 2020-08-31

**Authors:** I. Kostakis, R. Röttgers, A. Orkney, H. A. Bouman, M. Porter, F. Cottier, J. Berge, D. McKee

**Affiliations:** 1 Physics Department, University of Strathclyde, Glasgow, UK; 2 Remote Sensing Department, Helmholtz-Zentrum Geesthacht, Geesthacht, Germany; 3 Department of Earth Sciences, University of Oxford, Oxford, UK; 4 Scottish Association for Marine Science, Oban, UK; 5 Department Arctic and Marine biology, Faculty for Bioscience, Fisheries and Economy, UiT The Arctic University of Norway, Tromsø, Norway; 6 Department of Arctic Biology, University Center on Svalbard, Longyearbyen, Norway; 7 Department of Biology, NTNU AMOS—Center of Autonomous Marine Operations and Systems, Norwegian University of Technology and Science, Trondheim, Norway

**Keywords:** bio-optical model, Arctic Ocean, autonomous observations, ocean colour remote sensing, light availability

## Abstract

A bio-optical model for the Barents Sea is determined from a set of *in situ* observations of inherent optical properties (IOPs) and associated biogeochemical analyses. The bio-optical model provides a pathway to convert commonly measured parameters from glider-borne sensors (CTD, optical triplet sensor—chlorophyll and CDOM fluorescence, backscattering coefficients) to bulk spectral IOPs (absorption, attenuation and backscattering). IOPs derived from glider observations are subsequently used to estimate remote sensing reflectance spectra that compare well with coincident satellite observations, providing independent validation of the general applicability of the bio-optical model. Various challenges in the generation of a robust bio-optical model involving dealing with partial and limited quantity datasets and the interpretation of data from the optical triplet sensor are discussed. Establishing this quantitative link between glider-borne and satellite-borne data sources is an important step in integrating these data streams and has wide applicability for current and future integrated autonomous observation systems.

This article is part of the theme issue ‘The changing Arctic Ocean: consequences for biological communities, biogeochemical processes and ecosystem functioning’.

## 1. Introduction

The Arctic Ocean ecosystem is undergoing rapid changes in response to anthropogenic climate change, warming approximately twice as fast as the global average [1]. As a result, sea ice is declining in extent and volume [2], open water areas are increasing [3], enhanced river inflow is leading to an increase in fresh water [4] and lithogenic and biogenic fluxes are increasing due to thawing permafrost and glacial/river runoff [5,6].

Changes in physical conditions and material composition have implications for primary production, the marine food web and the global carbon cycle. The loss of sea ice results in an increase in light availability in the surface layer [7] and changing primary production [8–10]. Freshening of the surface Arctic also leads to changes in upper ocean stratification and nutrient supply [11] that has been shown to impact phytoplankton community structure [12]. Changes in phytoplankton abundance and composition (i.e. particle size) affect the biological carbon pump and, hence, the global carbon cycle in which the Arctic plays an important role [13].

Monitoring changes in Arctic primary productivity from ocean colour remote sensing has been undertaken previously [14,15], but is subject to several limiting factors. Extensive and persistent cloud cover severely limits the availability of cloud-free scenes to work with [16]. High latitudes impose generally low solar zenith angles which challenge the performance of standard atmospheric corrections and product retrieval algorithms [17–19]. Shipboard operations are particularly difficult in this remote region and under often harsh weather conditions, resulting in a relatively limited amount of *in situ* validation data having been collected in the region to date [14,20].

Knowledge of the optical properties of the Arctic Ocean is crucial to improve our understanding and predictions of changes in underwater and water-leaving light fields, and their impact on Arctic biogeochemistry. Key to this understanding is the development of (regional) bio-optical models that relate inherent optical properties (IOPs) (absorption, scattering and backscattering) to concentrations of optically significant water constituents such as phytoplankton, other organic particles, sediment and coloured dissolved organic material (CDOM). Existing bio-optical models are restricted to relatively small spatial and temporal scales, e.g. [21], and further effort is required to explore natural variability across the region. Establishment of robust bio-optical models enables exploitation of radiative transfer models that can be used to predict light availability for primary production and generation of ocean colour remote sensing reflectance signals.

The development of autonomous underwater sampling platforms has radically transformed our ability to make continuous observations in harsh environments [22–25]—although, to date, gliders and bio-Argo floats have rarely been used in the Arctic [26,27]. Although the sensor payload of these autonomous platforms varies, in many cases, they are equipped with a CTD and a triplet optical sensor measuring chlorophyll fluorescence, CDOM fluorescence and backscattering, with sensor outputs assigned as proxies for optical constituents with associated biogeochemical significance, e.g. chlorophyll fluorescence or particulate backscattering as an indicator of phytoplankton biomass. There are a number of challenges in the interpretation of such data, but there is also huge value in exploiting these datasets to a greater extent than has previously typically been the case. Autonomous platforms, predominantly bio-Argo floats, have been used to compare satellite retrievals of chlorophyll and particulate backscattering [28] with *in situ* measurements. Several studies have investigated the correlation between surface and total water column observations in different regions, such as the Southern Ocean [29] or the North Atlantic [30]. In this paper, we aim to develop a bio-optical model for the Barents Sea region of the Arctic Ocean that will be designed to use optical triplet data as proxy inputs for biogeochemical variables. By transforming *in situ* triplet data into estimates of bulk IOPs, we aim to create new means of accurately modelling underwater light fields, which are required to estimate primary productivity, based on *in situ* glider data. Very little light field data are available for the Arctic Ocean, especially in polar night when the region is not accessible and light levels are below the detection threshold of commonly available sensors. The combination of glider observations and bio-optical models can provide new insights and improve our understanding of the transition of the Arctic Ocean ecosystem and Arctic productivity from winter to spring. Results will further link *in situ* glider data to remote sensing reflectance consistent with ocean colour satellite observations. This is a practical implementation of an ambition to quantitatively link autonomous vehicle and remote sensing platform technologies that is widely stated, but seldom achieved in practice (see [31] for an example of the inverse of our approach).

We present a bio-optical model for the Barents Sea across three seasons (winter–summer) based on shipboard observations and discuss the pragmatic compromises required in order to achieve sensible performance using salinity, fluorescence and backscattering data measured by standard sensors deployed on autonomous vehicles as driving inputs. Commonly, difficult operating conditions affect data collection at sea, particularly in the Arctic. Here, we therefore present an approach that necessarily involved making pragmatic decisions about how to best exploit the limited data available. Where possible, we have used other established optical datasets (e.g. NOMAD) to inform our analysis and to provide broader context for the fitting of relationships. The performance of the approach is assessed by comparison with shipboard *in situ* optical profiles taken at the start and end of extended glider deployments, and through comparison with ocean colour imagery taken during the course of a glider deployment. We will show that the ability to express glider data as bulk IOPs provides new opportunities to gain new insights into seasonal changes in the underwater light field, timing and location of primary production, and relationships with ocean colour remote sensing signals.

## 2. Methods

### (a) The dataset

#### (i) Fieldwork location and timing

Data for this study were collected in the Barents Sea from January to July 2018, during three different research cruises and two glider deployments. The Barents Sea is a relatively shallow continental shelf sea with an average depth of 230 m and maximum of 500 m. Its hydrography is influenced by Atlantic-derived, relatively warm and saline water entering from the Southwest and colder Arctic-derived water in the Northeast.

Details on research vessels, cruise timing, sample locations and glider deployments can be found in table 1.

**Table 1.** Summary of sampling activities during three cruises in the Barents Sea in 2018.

	winter	spring	summer
sampling period	5 Jan–17 Jan 2018	26 April–4 May 2018	12 June–4 July 2018
sampling area	70.045 °N, 015.800 °E–	74.616 °N, 014.997 °E–	74.366 °N, 009.474 °E–
	78.193 °N, 030.057 °E	78.599 °N, 030.083 °E	82.592 °N, 031.344 °E
research vessel	Helmer Hanssen	Helmer Hanssen	James Clark Ross
max. no. sample absorption	7	9	34
no. *in situ*	6	4	—
IOP profiles			
glider 1	*deployed:*	*recovered*:	—
	11 January 2018	20 April 2018	
	76.50 ° N, 029.00° E	75.10° N, 030.13° E	
glider 2	—	*deployed*:	*recovered*:
		26 April 2018,	4 June 2018
		74.62° N, 029.84° E	76.86° N, 029.90° E
satellite image	—	MODIS-A 8 day average:	—
		30 April–8 May 2018	
chlorophyll concentrations	—	1.5–15.6 mg m^−3^	0.3–11.3 mg m^−3^
total suspended matter concentration	0.2–1.1 mg l^−1^	1.1–8.8 mg l^−1^	0.3–15.2 mg l^−1^
*a*_CDOM_(440)	0.036–0.070 m^−1^	0.031–0.050 m^−1^	0.027–0.054 m^−1^

#### (ii) Water sample analyses

Chlorophyll *a* concentration, *Chl*, of water samples was determined using a Turner Designs Trilogy fluorometer calibrated in between cruises using chlorophyll *a* spinach standard (Sigma Aldrich), showing negligible drift. *Chl* was determined in triplicate by filtering 200 ml of water sample onto GF/F filters, which were subsequently immersed in 10 ml of 90% acetone solution and stored in the dark at −20°C overnight. Fluorescence was measured the following morning, before and after acidification with 10% HCl solution [32]. *Chl* ranges for the different seasons are given in table 1.

**Figure 1.**
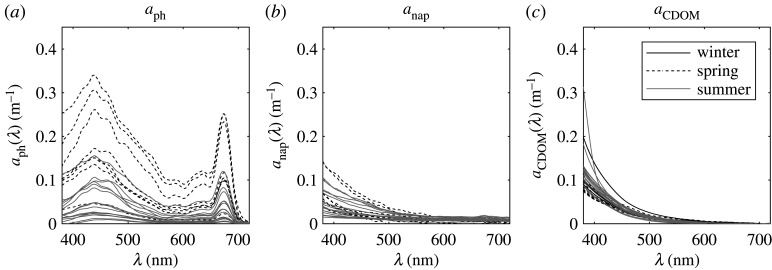
(*a*) Phytoplankton, (*b*) non-algal particulate and (*c*) CDOM absorption spectra measured in the Barents Sea in winter (Jan), spring (Apr) and summer (Jun).

Absorption of coloured dissolved organic matter (CDOM), *a*_CDOM_, was measured using a liquid waveguide capillary system (LWCC) with a 1 m pathlength (see [33] for details on the set-up, components and methods). In short, samples were filtered through 0.2 µm pore size syringe filters while being injected into the capillary cell using a peristaltic pump. Measurements were made against purified water (Milli-Q) and corrected for salinity and temperature effects on the absorption by pure water. Spectra were smoothed using a 5-point moving average and offset corrected at 700 nm (figure 1*c*).

Spectral particulate absorption coefficients, *a_p_*, were determined following the methods described in [34]. The optical density (OD) of particles freshly filtered onto a GF/F filter was measured with a dual-beam spectrophotometer (Shimadzu, UV-2501-PC) against blank reference filters which had been rinsed with filtered seawater. Measurements were set up to record a single scan from 300 to 800 nm. Pigments were bleached using a few drops of a 10% sodium hypochlorite solution [35]. Filters were rinsed with filtered seawater and scanned again to determine the absorption by non-algal particulate matter, *a*_nap_. OD (both total particulate and non-algal) was converted into absorption coefficients, *a_p_* (figure 1*a*) and *a*_nap_ (figure 1*b*), using a linear regression approach [34]. This correction method compares filter pad measurements against particulate absorption determined by subtracting CDOM absorption measured with an LWCC system from total non-water absorption measured in a Point-Source Integrating-Cavity Absorption Meter (PSICAM, [36]). PSICAM measurements had been calibrated daily using a Nigrosine solution and a corresponding absorption coefficients measurement with an LWCC system. Finally, phytoplankton absorption spectra, *a*_ph_, were calculated as difference between *a_p_* and *a*_nap_.

#### (iii) *In situ* optical and hydrographic profiling

**Figure 2.**
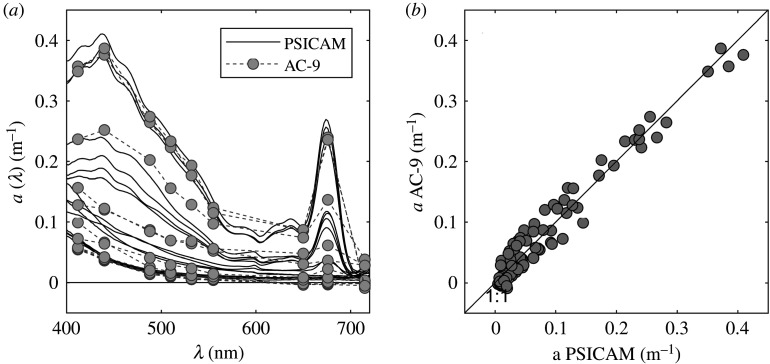
Comparison between PSICAM and AC-9 absorption. (*a*) Absorption spectra of water samples taken from the top 15 m in January and April 2018. (*b*) Corrected AC-9 versus PSICAM absorption at 9 AC-9 wavelengths. All AC-9 data have been corrected using the iterative correction (McKee *et al*. [38]). At some January stations, absorption coefficients at wavelengths greater than 600 nm were negative, challenging instrument sensitivity in these very clear waters.

**Figure 3:**
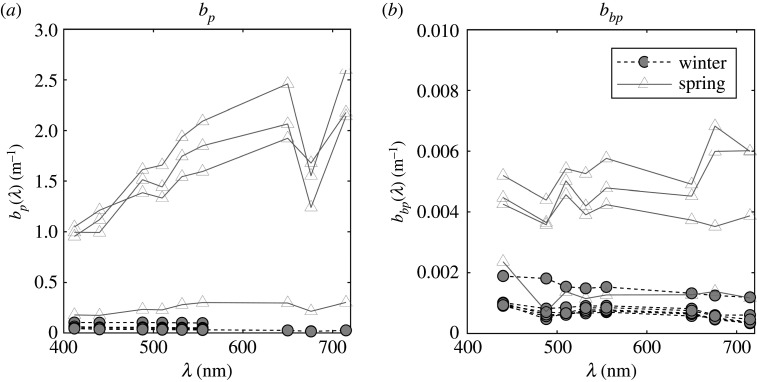
(*a*) Scattering and (*b*) backscattering spectra measured in the Barents Sea in winter and spring. Scattering spectra have been derived from AC-9 measurements corrected using the iterative correction. Winter scattering coefficients corresponding to negative absorption values (figure 2) not shown.

Profiles of *in situ* non-water absorption, *a*_nw_, and attenuation, *c*_nw_, were measured at nine wavelengths using an AC-9 (WET Labs Inc.) equipped with 25 cm cuvettes during the winter and spring cruises (no profiling optical data could be collected for the summer cruise). AC-9 measurements were calibrated using purified water in the laboratory before the first cruise and later during cruises on-board the ship. Average measured offsets were subtracted from absorption and attenuation spectra in post-cruise processing [37]. Data were further corrected for the effects of salinity and temperature using simultaneously collected CTD data (Seabird, SBE19Plus v. 2). In addition, data were corrected for scattering errors using the iterative correction approach [38]. The performance of the scattering correction was tested by comparing corrected AC-9 absorption with PSICAM absorption obtained from samples collected at the same depth (figure 2). Scattering spectra were calculated by subtracting absorption from attenuation (figure 3*a*). It is important to note that, in the clearest waters (in January), absorption signals were very low, close to the limit of sensitivity of the AC-9, with some samples returning negative absorption values that have been omitted from further analysis (including calculation of scattering coefficients).

Profiles of *in situ* volume scattering function at 124° were measured at 9 wavelengths with a BB9 (WET Labs Inc.) mounted alongside the AC-9 and CTD. BB9 wavelengths were linearly interpolated to match AC-9 wavelengths and converted to backscattering coefficients following the manufacturer's manual [39], using a *χ*-factor of 1.076 [40] and data from the AC-9 to correct for pathlength absorption effects (figure 3*b*). Backscattering data at 412 nm were excluded from all analysis due to known issues with data quality and drift of the channel.

Hydrographic measurements were made using a Seabird SBE19Plus v2 CTD equipped with conductivity, temperature and pressure sensors which was mounted on the same frame as *in situ* IOP instruments. The instrument was calibrated by the manufacturer before the start of sampling.

#### (iv) Glider deployment

Two shallow G2 Slocum gliders were deployed as part of this campaign, keeping near continuous occupation of a north–south transect along 30° E between January and June 2018. Both gliders were equipped with a pumped CTD sensor (Seabird, 41) and a triplet ECO-sensor (WET Labs, Inc.) measuring backscattering at 700 nm, chlorophyll fluorescence and CDOM fluorescence. The glider which was in operation from April–June was also equipped with a *PAR* sensor. The data were collected in a sawtooth pattern, typical of a glider diving profile and separated into individual profiles before being checked for data spikes. Corrections were calculated for the data from calibration profiles collected by the ship during the glider deployment. Due to unexpected failures towards the end of each glider mission, it was not possible to collect end-of-mission profiles for calibration purposes. The de-spiked data were then separated into individual north–south transects and gridded following a Barnes objective analysis method, with grid spacings of 2 km × 5 m [41].

#### (v) Ocean colour remote sensing data

Remote sensing reflectance spectra were extracted from an 8-day average MODIS-A image (standard processing) covering the period of 30 April–8 May 2018. Temporal averaging (of at least 8 days) was required to achieve good spatial coverage across the Barents Sea. As a result, satellite data were used for broad comparison with our bio-optical model rather than one-to-one match-ups with individual scenes. It should also be noted that the development of the spring bloom can change the optical properties of the surface layer in the Arctic Ocean extremely rapidly (within days) further limiting the feasibility of direct comparisons with average satellite images.

### (b) Bio-optical model development

Our observations suggest that the optical properties of the Barents Sea are largely driven by phytoplankton and related detrital materials and CDOM. We propose a bio-optical model which relates all particle properties to *Chl* as a proxy for phytoplankton biomass, and absorption by CDOM is related to salinity, *Sal*, reflecting the assumption that freshwater inflow is the dominant source. Total absorption, *a*, scattering, *b*, and backscattering, *b_b_*, coefficients can be expressed as the sum of all partial IOPs, i.e. the IOPs associated with each relevant optically significant component

2.1 $$\begin{array}{rl} a(\lambda ) & =a_{w}(\lambda )+a_{\text{CDOM}}(\lambda ,\text{Sal})+a_{\text{ph}}(\lambda ,\text{Chl})+a_{\text{nap}}(\lambda ,\text{Chl})({\text{m}}^{-1}), \end{array}$$

2.2 $$\begin{array}{rl} b(\lambda ) & =b_{w}(\lambda )+b_{p}(\lambda ,\text{Chl})({\text{m}}^{-1}) \end{array}$$

2.3 $$\begin{array}{rl} \;\text{and}\;b_{b}(\lambda ) & =b_{\text{bw}}(\lambda )+b_{\text{bp}}(\lambda ,\text{Chl})({\text{m}}^{-1}), \end{array}$$

where the subscripts *w*, ph, nap, CDOM and *p* refer to water, phytoplankton, non-algal particles, coloured dissolved organic material and particles, respectively. Absorption, scattering and backscattering coefficients of seawater are well described in the literature [42–45].

In the following sections, we attempt to derive appropriate models for each of the partial IOPs mentioned in equations (2.1)–(2.3). As shall be seen, the quantity of data we were able to generate for some of the parameters was limited, constrained by difficult sampling conditions and limited time available at sea. In many respects, this is representative of modern sampling strategies where sensors on autonomous platforms are expected to carry an increasing share of the sampling burden. In what follows, we outline the necessary pragmatic steps required to provide suitable bio-optical relationships that can be used to convert *in situ* glider observations to bulk optical properties, with only limited local *in situ* data available.

#### (i) Partial inherent optical properties: absorption

**Figure 4.**
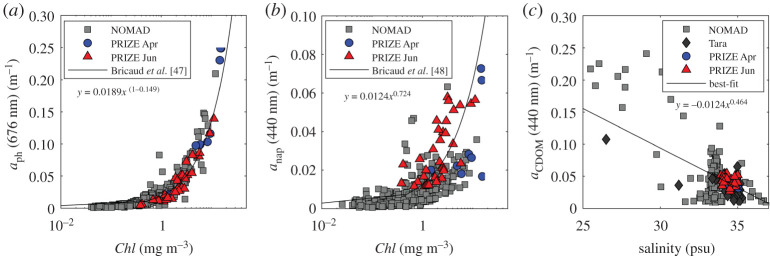
(*a*) *a*_ph_ and (*b*) *a*_nap_ plotted against *Chl*, and (*c*) *a*_CDOM_(440) plotted against salinity, *Sal.* Coloured symbols are data from the Barents Sea measured in spring and summer. Grey symbols represent the global NOMAD dataset, dark grey diamonds are Tara data and solid lines show bio-optical relationships. (Online version in colour.)

Figure 4*a*,*b* shows *a*_ph_ and *a*_nap_ plotted against *Chl* for Barents Sea data collected for this study. To provide wider context, associated results from the global NOMAD dataset [46] have also been plotted in these figures. The volume of data available from our Barents Sea dataset alone would limit confidence in production of robust local bio-optical relationships. However, our observations were found to be consistent with the global NOMAD dataset and relationships for algal and non-algal absorption were well described by existing literature relationships [47,48] (equations (2.4)–(2.6))

2.4 $$a_{\text{ph}}(\lambda ,\text{Chl})=A_{\text{ph}}(\lambda ){\text{Chl}}^{\left(1+B_{\text{ph}}(\lambda )\right)}({\text{m}}^{-1}),$$

with coefficients *A*_ph_ and *B*_ph_ from Bricaud *et al.* [41].

2.5 $$a_{\text{nap}}(\lambda ,\text{Chl})=a_{\text{nap}}(440)\exp[-0.011(\lambda -440)]({\text{m}}^{-1})$$

and

2.6 $$a_{\text{nap}}(440)=0.0124\;\text{Ch}{\text{l}}^{0.724}({\text{m}}^{-1}),$$

Previous studies have shown that CDOM absorption does not covary with seasonal changes in *Chl* in the Barents Sea [49,50]. In coastal environments and the Arctic shelf, CDOM absorption exhibits a strong relationship with salinity, decreasing with increasing salinity [51,52]. Matsuoka *et al*. [53] derived a relationship between salinity, *Sal*, and *a*_CDOM_(440) for the Arctic waters of the South Beaufort Sea. However, these relationships are strongly driven by coastal waters with high CDOM absorption and low salinity and tend to underestimate CDOM for *Sal *> 28 PSU, as typically observed in the Barents Sea (33–35 PSU in this study). We, therefore, derived a new relationship to link *a*_CDOM_(440) to *Sal* based on our Barents Sea data as well as additional pan-Arctic data [54] with salinity ranging from 27 to 35 PSU (figure 4*c*)

2.7 $$a_{\text{CDOM}}(440)=[-0.012(\pm 0.003)\text{Sal }+0.464(\pm 0.183)]({\text{m}}^{-1}).$$

The spectral shape was modelled using equation (2.8) and a slope of −0.0168 nm^−1^, the median slope of all CDOM absorption spectra we measured in the Barents Sea. It was considerably lower than the average of −0.0187 which has been observed in other Arctic waters [55]

2.8 $$a_{\text{CDOM}}(\lambda ,\text{Sal})=a_{\text{CDOM}}(440)\exp[-0.0168(\lambda -440)]({\text{m}}^{-1})$$

#### (ii) Partial inherent optical properties: scattering and backscattering

**Figure 5.**
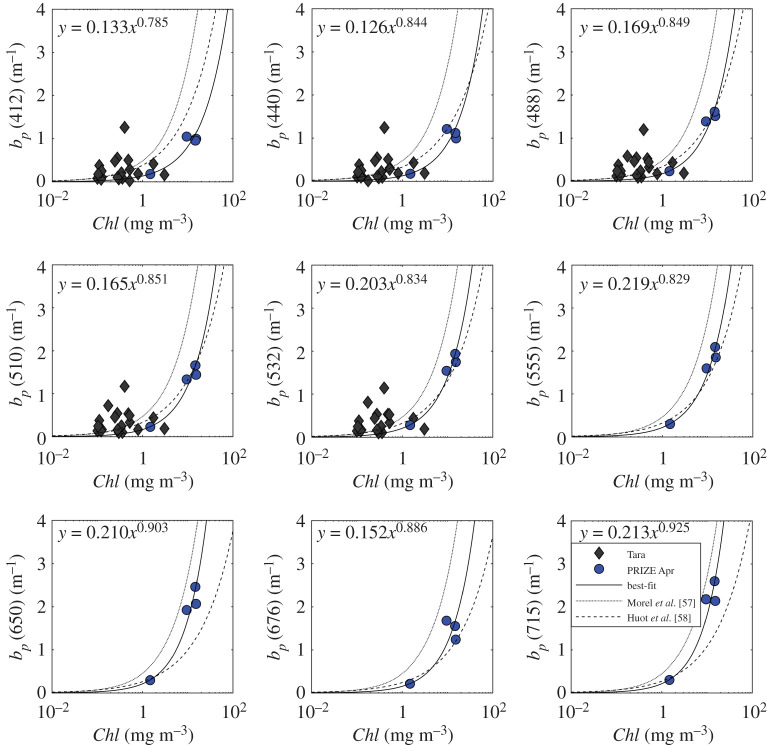
Relationship between *b_p_* and *Chl* at nine different wavelengths. Dotted and dashed lines show the relationships by Morel *et al*. [57] and Huot *et al*. [58], respectively. The solid lines are best-fit power laws through the available Barents Sea data. (Online version in colour.)

Little spectral *b_p_* data have been published, possibly reflecting concerns about the ability to correct corresponding attenuation measurements for variable collection angle errors [38,56]. Despite best efforts, the combination of no optical profiles in summer and winter *Chl* levels below detection limits meant that only the four *b_p_* spectra collected in the Barents Sea during the spring cruise were available for model development. Comparison with other scattering data collected across a range of Arctic waters in summer and autumn [54] showed, as expected, that chlorophyll and scattering are higher in the Barents Sea in spring, when productivity is at its maximum. Established relationships by Morel *et al*. [57] and Huot *et al*. [58] were compared to the observations. The Morel *et al*. relationship which was developed for open waters was found to consistently overestimate Barents Sea *b_p_* (figure 5). The model by Huot *et al*. [58] matched our data well at blue and green wavelengths but underestimated *b_p_* at red/NIR wavelengths. Therefore, following the approach presented in [58], wavelength-dependent relationships between *Chl* and *b_p_* were derived by fitting a power-law to the available Barents Sea data, using a least-square linear regression applied to the log-transformed dataset with subsequent conversion back into linear space (equation (2.9), table 2). Normally, we would hesitate to apply such regression to only four data points. The approach we present here is a pragmatic attempt to maximize exploitation of the limited data that are available and represents a sensible response to a common problem when working with autonomous platforms, where *in situ* validation data are typically only collected at the beginning and end of a deployment. The resulting relationship takes the form

2.9 $$b_{p}(\lambda ,\text{Chl})=A_{b}(\lambda )\text{Ch}{\text{l}}^{\left({B_{b}}_{(}\lambda )\right)}({\text{m}}^{-1}),$$

with the regression coefficients *A_b_* and *B_b_* given in table 2.

**Table 2.** Scattering model coefficients (see equation (2.9)), associated 95% confidence intervals and coefficient of determination.

	*A_b_*	*B_b_*	*R*^2^
412	0.133 (± 1.768)	0.785 (± 0.255)	0.9457
440	0.126 (± 2.044)	0.844 (± 0.320)	0.9265
488	0.169 (± 1.449)	0.849 (± 0.166)	0.9802
510	0.165 (± 1.451)	0.851 (± 0.167)	0.9801
532	0.203 (± 1.365)	0.834 (± 0.139)	0.9855
555	0.219 (± 1.326)	0.829 (± 0.126)	0.9879
650	0.210 (± 1.507)	0.903 (± 0.184)	0.9786
676	0.152 (± 2.311)	0.886 (± 0.375)	0.9089
715	0.213 (± 1.685)	0.925 (± 0.233)	0.9671

**Figure 6.**
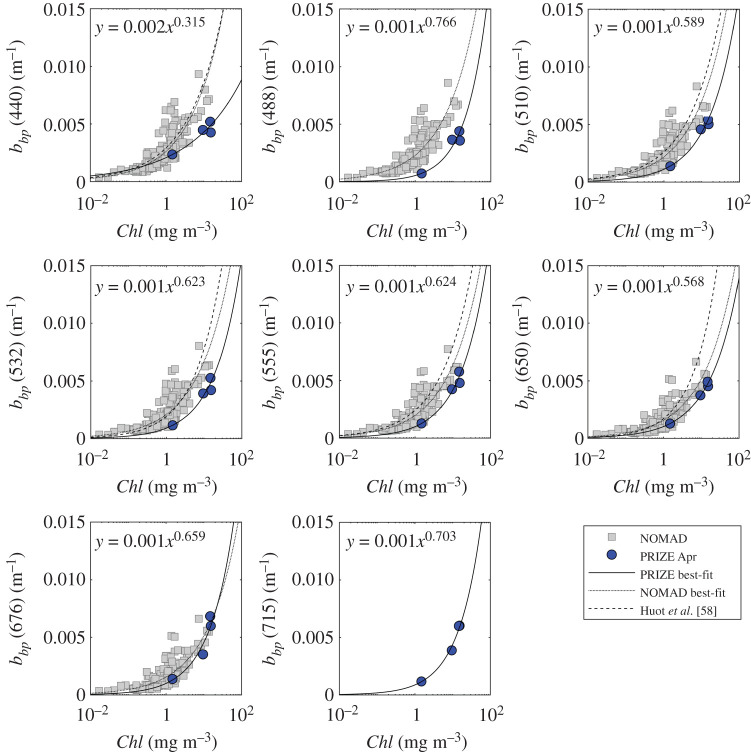
Relationship between *b*_*bp*_ and *Chl* at eight different wavelengths. The dotted line shows the NOMAD best-fit, the dashed line the relationship by Huot *et al*. [58] and the solid line is the best-fit power law through available Barents Sea data. (Online version in colour.)

Figure 6 shows particulate backscattering, *b_bp_*, plotted against *Chl* for both the Barents Sea and the global NOMAD datasets. Barents Sea *b_bp_* data appear to be at the low end of the distribution for the NOMAD dataset. The relationship between *b_bp_* and *Chl* described by Huot *et al*. [58] (dashed line figure 6) and the best-fit to the NOMAD dataset (dashed line figure 6) both tend to overestimate Barents Sea *b_bp_*. Using an approach analogous to that used for *b_p_* above, a set of spectral coefficients *A_bb_* and *B_bb_* were derived to describe the relationship between *Chl* and *b_bp_* (table 3, equation (2.10)). Again, it is necessary to recognize that such a small number of data points provides a limited basis for deriving such relationships. However, in this case, comparison with the NOMAD dataset provides a degree of comfort that the overall structure is broadly similar

2.10 $$b_{bp}(\lambda ,\text{Chl})=A_{bb}(\lambda ){\left[\text{Chl}\right]}^{\left(B_{bb}(\lambda )\right)}({\text{m}}^{-1}).$$

**Table 3.** Backscattering model coefficients (see equation (2.10)), associated 95% confidence intervals and coefficient of determination.

	*A_bb_*	*B_bb_*	*R*^2^
440	0.002 (± 1.312)	0.315 (± 0.121)	0.9243
488	0.001 (± 1.555)	0.766 (± 0.198)	0.9656
510	0.001 (± 1.237)	0.589 (± 0.095)	0.9865
532	0.001 (± 1.366)	0.623 (± 0.140)	0.9740
555	0.001 (± 1.290)	0.624 (± 0.114)	0.9827
650	0.001 (± 1.128)	0.568 (± 0.054)	0.9953
676	0.001 (± 1.569)	0.659 (± 0.202)	0.9519
700^a^	0.001 (± 1.313)	0.683 (± 0.122)	0.9835
715	0.001 (± 1.178)	0.703 (± 0.073)	0.9835

^a^Extrapolated/interpolated.

### (c) Converting glider observations to chlorophyll concentration

The bio-optical model described above uses *Chl* and *Sal* as currency parameters, both routinely measured by standard sensors on gliders and other autonomous platforms. *Sal* is straightforwardly derived from the conductivity measurement of a CTD. *Chl* can be estimated from the chlorophyll fluorescence measurement of the WET Labs ECO sensor (excitation: 470 nm, emission: 695 nm). However, the accuracy of *Chl* estimates based on fluorescence measurements is known to be limited due to the effects of fluorescence quenching in the surface layer and other physiological processing impacting fluorescence efficiency [59–61]. Recently published correction methods [61,62] require either simultaneous *PAR* measurements and/or dark values. Unfortunately, logistical constraints meant that *PAR* sensors were not operational on all of the gliders. In addition, it is necessary to cross-calibrate fluorescence-based *Chl* estimates from manufacturer's laboratory calibrations against concentration measurements of water samples collected simultaneously. Unfortunately, again due to logistical constraints, cross-calibration measurements were only available for the beginning of each glider deployment. Missing calibration data at the end of each glider deployment means that measurements cannot be corrected for potential drift or bio-fouling of the sensors.

**Figure 7.**
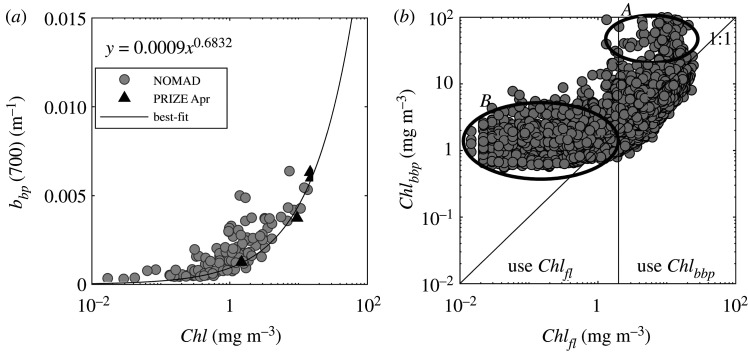
Conversion of glider backscattering at 700 nm to chlorophyll concentration. (*a*) *b_bp_* from NOMAD dataset (extrapolated to 700 nm), Barents Sea data (interpolated to 700 nm) and best-fit relationship to PRIZE data (equation (2.10), table 3). (*b*) Chlorophyll concentration estimated from glider backscattering measurements, *Chl*_*bbp*_, compared to chlorophyll estimated from fluorescence measurements, *Chl*_*fl*_. Area *A* highlights fluorescence measurements potentially affected by solar quenching while area *B* shows the range in which the backscattering sensor shows limited dynamic range.

The challenge, therefore, was twofold: (i) Can we establish a method of validating the fluorescence-based estimate of *Chl* from the triplet puck? (ii) Can we estimate *Chl* from glider data free from solar quenching issues near the surface during daylight hours? The solution to these issues is to consider the relationship between backscattering from the triplet sensor and *Chl*. It has already been established (above) that particulate IOPs are well correlated with *Chl* for these waters. Backscattering measurements, although still susceptible to bio-fouling of the sensor, are not affected by fluorescence quenching. The glider triplet sensors measured backscattering at 700 nm (not one of the AC-9/BB9 wavelengths) resulting in the requirement for deriving an additional relationship, linking *b_bp_* (700) to *Chl* (figure 7*a*). As NOMAD *b_bp_* data are limited to less than 683 nm, spectra were extrapolated to 700 nm using a power-law relationship. Barents Sea BB9 data were linearly interpolated to 700 nm and were very consistent with NOMAD observations. For consistency, a power-law was fitted to Barents Sea backscattering data only (see above). This relationship was subsequently used to convert *b_bp_* (700) to *Chl* (figure 7*a* and table 3).

Discrepancies between *Chl* derived from backscattering measurements, *Chl*_*bbp*_, and fluorescence measurements, *Chl*_*fl*_, increased for very low and very high chlorophyll concentrations (figure 7*b*). At high *Chl*, *Chl*_*bbp*_ tended to be higher than *Chl*_*fl*_ (area *A*, figure 7*b*). This was attributed to the fact that *Chl*_*fl*_ is likely to underestimate true chlorophyll concentration due to fluorescence quenching. High *Chl* values are found in surface waters in this region and at this time of year (see later). At low chlorophyll concentrations, *Chl*_*bbp*_ was found to be higher than *Chl*_*fl*_ which could potentially be due to unresolved background particles (not associated with *Chl*), a residual offset in the *b_bp_*-to-*Chl* relationship, or limitations in the dynamic range of the backscattering sensor (area *B*, figure 7*b*). Given this limitation, we, therefore, developed a combined approach for this work, in which *Chl* was derived from the fluorescence measurements up to a concentration of 2 mg m^−3^. For *Chl*_*fl* _> 2 mg m^−3^, *Chl* was estimated based on the backscattering measurement and the relationship obtained from figure 7*a*. This relatively convoluted approach reflects real world bio-optical complexity and potential sensor performance limitations, but is easy enough to implement and could be transferred to other areas with minimal local sampling. It should be noted, however, that the relationship is driven by spring backscattering data and is potentially a poor representation of winter *b_bp_* spectra (details discussed below).

## 3. Results

### (a) Performance test of bio-optical model

**Figure 8.**
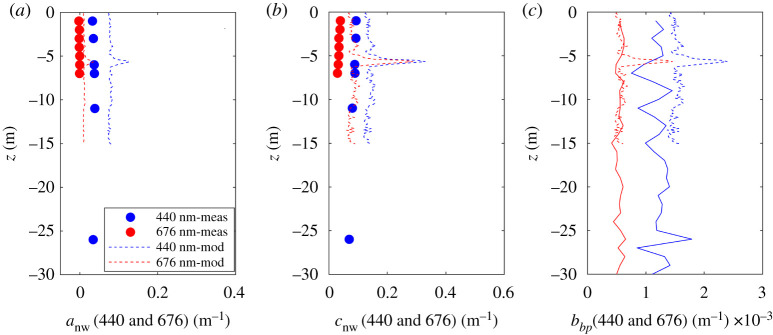
Comparison between measured (solid) and modelled (dashed) IOP profiles at 440 and 676 nm. Measurements were made on 11 January 2019, 14.24, at 76.5057 N 029.1188 E. Modelled profiles are based on glider measurements of *Chl*_*fl*_, *b_bp_*(700) and *Sal* made on 11 January 2019, 17.06, at 76.4930 N 029.0267 E. (Online version in colour.)

**Figure 9.**
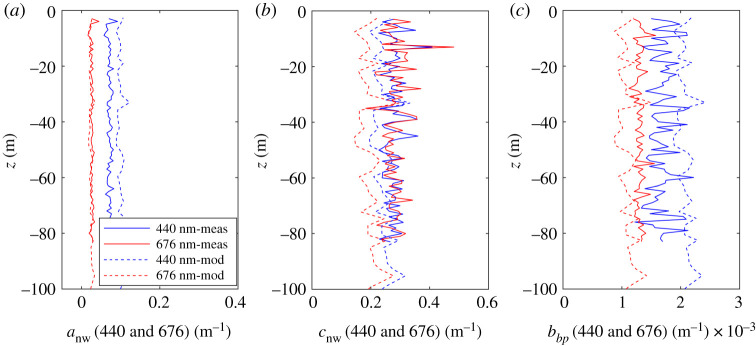
Comparison between measured (solid) and modelled (dashed) IOP profiles at 440 and 676 nm. Measurements were made on 26 April 2019, 06.30, at 74.6157° N 030.0547° E. Modelled profiles are based on glider measurements of *Chl*_*fl*_ and *b_bp_*(700) and *Sal* recorded on 27 April, 13.30, at 74.7558° N 030.0473° E. (Online version in colour.)

A set of *in situ* IOP profiles was measured in close proximity to each of the glider deployment sites in January (figure 8) and April (figure 9). To test the performance of the bio-optical model, IOPs were modelled based on the first triplet/CTD profile obtained during each glider deployment and subsequently compared to the *in situ* IOP measurements. Non-water absorption, non-water attenuation and particulate backscattering coefficient profiles were compared at two wavelengths, 440 and 676 nm. Average deviation of modelled and measured *a*_nw_ and *b_bp_* January profiles for both wavelengths were 0.028 m^−1^ (greater than 100%) and +0.0004 m^−1^ (22%), respectively. Modelled *c*_nw_ tended to overestimate measured values by on average 0.059 m^−1^ (68% at 440 nm, greater than 200% at 676 nm). IOPs in winter were extremely low, resulting in large relative deviations between modelled and measured data which can be attributed to uncertainties in both the *in situ* measurements and model performance.

In April, when Barents Sea waters have become more productive, glider estimates are more consistent with *in situ* measurements both relatively and in absolute magnitude, with average deviations of 0.012 m^−1^ (22%), −0.083 m^−1^ (24%, underestimation of *in situ* data) and 0.0001 m^−1^ (8%) for *a*_nw_, *c*_nw_ and *b_bp_*, respectively. In general, modelled IOP profiles exhibited increased noise compared to *in situ* measurements as a result of spikes in the glider *b_bp_* measurements.

### (b) Inherent optical property transects from glider data

**Figure 10.**
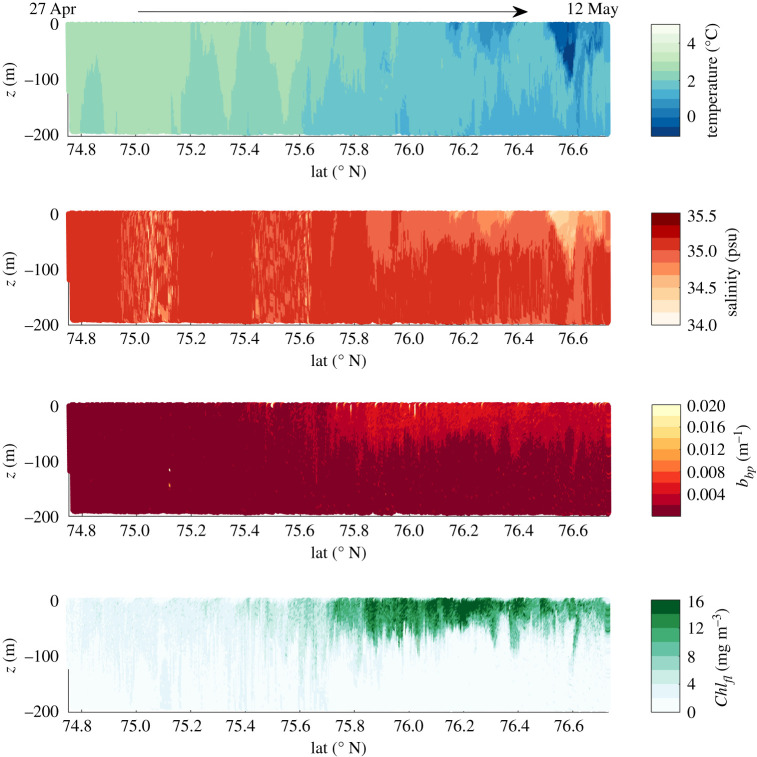
South-to-North glider transect of temperature, salinity, fluorescence-based chlorophyll estimates and *b_bp_* (700) measured in the Barents Sea, 27 April–12 May 2018. (Online version in colour.)

Figure 10 shows temperature, salinity, *Chl*_*fl*_ and *b_bp_* (700) data measured along the first South-to-North transect of the second glider deployment from 27 April into May. The glider crossed the polar front at approximately 75.8° N, transitioning from more Atlantic waters to Arctic waters. This change can be observed as a drop in water temperature and salinity as well as an increase in surface chlorophyll fluorescence and *b_bp_*, i.e. particle concentration, in the more stratified, productive Arctic waters.

**Figure 11.**
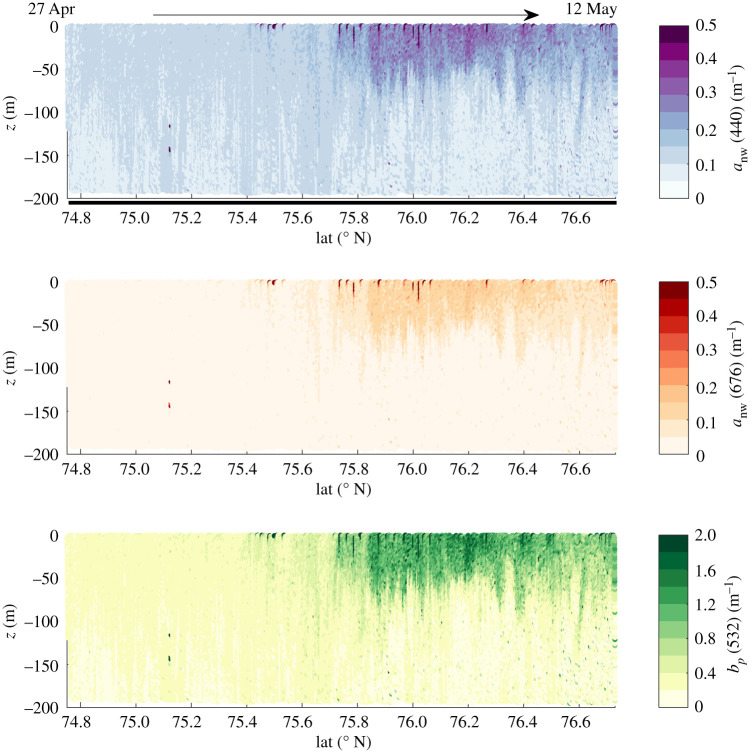
South-to-North transect of total non-water absorption, *a*_nw_, at 440 nm and 676 nm and particulate scattering, *b_p_*, at 532 nm modelled based on glider measurements of *b_bp_* and salinity in the Barents Sea, 27 April–12 May 2018. (Online version in colour.)

Based on the glider measurements of salinity, *b_bp_* and chlorophyll fluorescence, transects of absorption, scattering and backscattering coefficients were calculated at nine different wavelengths. IOPs increased in the top 50–100 m north of 75.8° N, corresponding to the gliders transition into Arctic waters with high surface biomass (figure 11).

### (c) Comparison with remote sensing reflectance spectra

Glider-based estimates of remote sensing reflectance spectra, *R*_*rs*_glider_, were calculated from the average of modelled *a* and *b*_*b*_ data within 2 m of sea surface using

3.1 $$R_{rs\_\text{glider}}=\frac{f}{Q}\frac{b_{b}}{a}\frac{1-r_{F}}{{\eta_{w}}^{2}}({\text{m}}^{-1}),$$

with *f/Q* = 0.0922 after Morel & Gentili [63], the approximate Fresnel reflectance of the surface as seen from the water side, *r_F_* = 0.021, and the refractive index of water, *η_w _*= 1.34 [64]. This simplified model does not include Raman scattering or bidirectional reflectance distribution function effects, potentially introducing significant bias at low solar zenith angles at high latitudes.

**Figure 12.**
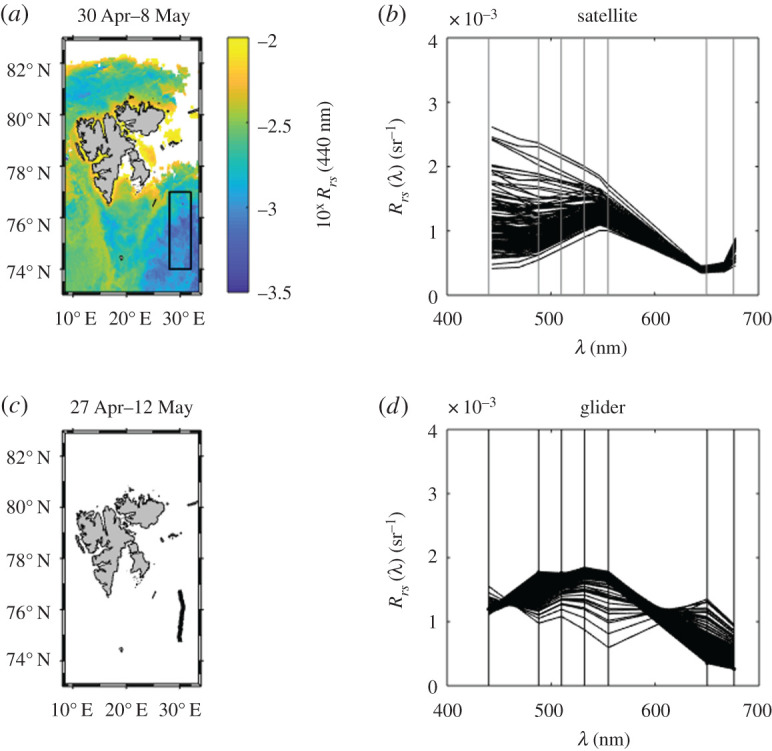
Comparison of *R*_*rs*_ spectra (*a*,*b*) obtained from MODIS-A 8-day average and (*c*,*d*) modelled based on optical triplet and salinity measurements from a glider. (Online version in colour.)

Modelled spectra (figure 12*d*) were compared to *R*_*rs*_ spectra obtained from MODIS-A satellite data (figure 12*b*), extracted from an 8-day average (inset box, figure 12*a*). The Arctic region is notoriously cloudy (IOCCG Report No. 16, 2015 [16]) and the 8-day average was required in order to present a reasonably complete image of the study region. The glider track shown in figure 12*c* represents an even longer period (16 days) and reflects the relatively slow lateral velocity of the glider. Water mass advection and changes in biological activity over this time period, together with variations in sampling times between the two platforms, make a pixel-to-transect direct comparison meaningless. We, therefore, avoid any attempt to perform a pixel-to-transect direct comparison and instead take a statistical approach.

**Figure 13.**
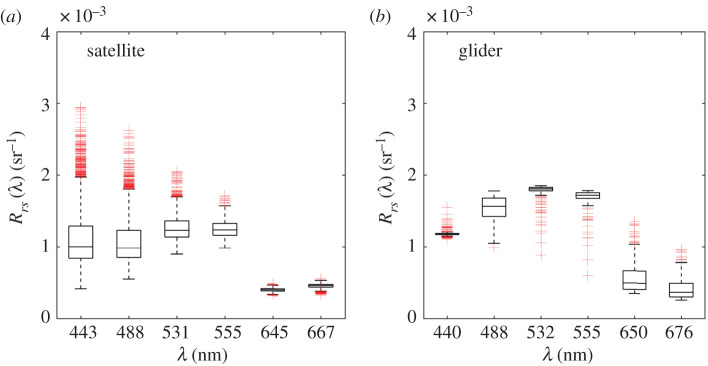
Boxplot of *R*_*rs*_ spectra from (*a*) obtained from MODIS-A 8-day average and (*b*) modelled based on *b_bp_* and *Sal* measured by a glider at closest matching wavebands (no interpolation). Plots show average values (horizontal lines), standard deviation and 95% confidence intervals as well as outliers (red ‘+’ marks) for each of the two datasets. (Online version in colour.)

On average, glider *R_rs_* satellite data has a mean error of 18% across all shared wavebands (figure 13). At 440/443 nm, satellite *R*_*rs*_ ranged from 0.41 × 10^−3^ sr^−1^ to 2.9 × 10^−3^ sr^−1^ and glider *R*_*rs*_ showed significantly less spread with values between 1.1 × 10^−3^ sr^−1^ and 1.6 × 10^−3^ sr^−1^. At red wavebands, satellite-derived *R*_*rs*_ was lower than glider *R*_*rs*_, with some *R*_*rs*_glider_ spectra showing peaks likely driven by features in *b_bp_.* An increase in *R*_*rs*_satellite_ from 645 to 676 nm potentially indicated the start of a sun induced chlorophyll fluorescence signal at this wavelength (not included in the bio-optical model). Overall, modelled *R*_*rs*_glider_ spectra were broadly comparable with satellite *R*_*rs*_ observations, with the exception of a few outlier spectra with ‘abnormal’ spectral shapes (figure 12*d*).

## 4. Discussion

The light environment in surface waters of the Barents Sea, and the Arctic Ocean in general, undergoes dramatic seasonal changes, from total darkness in winter to 24 h daylight during the summer months. *In situ* optical data of the region remain relatively scarce and are generally limited to light summer months. This has limited our understanding of bio-optical and associated biogeochemical processes within the marine Arctic ecosystem. Here, we collected a unique dataset of spectral IOP data on three cruises (winter, spring and summer of a single year) providing information on bio-optical relationships across different seasons. The resulting bio-optical model for the Barents Sea is potentially of interest for a variety of future studies including radiative transfer simulations of seasonally changing underwater light fields and resulting impacts on algal photosynthesis and underwater visibility. Here, however, we have focused on using the bio-optical model to demonstrate our ability to quantitatively link underwater glider observations to satellite ocean colour remote sensing signals. The approach enables us to take data from standard instruments (CTD for *Sal* and optical data from triplet sensors to derive *Chl*) and produce estimates of bulk optical properties that have been shown to be broadly consistent with results from *in situ* profiles, and that, in turn, are also broadly consistent with remote sensing reflectance signals.

The optical triplet instruments used in this study are regularly deployed on gliders and other autonomous vehicles. We believe that this is a valuable data resource that has been underexploited to date. One of the limiting factors has been concern over the interpretation of chlorophyll fluorescence signals due to potential issues in manufacturer's calibrations and effects of solar quenching during daylight hours in surface waters. Recent studies have proposed different correction methods for chlorophyll fluorescence measurements, using day/night comparisons, dark measurements at great depth for offset correction or simultaneous *PAR* measurements for quenching correction, or a combination [54]. Unfortunately, most existing approaches were not applicable for the glider deployments in this study due to the absence of these auxiliary data and day/night patterns. The approach we have developed offers a pathway to mitigate quenching effects using the backscattering data that these sensors simultaneously generate. Note that the switching threshold between *Chl*_*bbp*_ and *Chl*_*fl*_ implemented in this study would need to be adapted for operations elsewhere, but the principle is generally transferrable. The empirical relationship to calculate *Chl*_*bbp*_ is potentially a significant source of uncertainty in our bio-optical model, highlighting the need for a fluorescence quenching correction for deployments at high latitudes, in particular on relatively shallow continental shelfs. Inadequate calibration of optical sensors is, of course, a fundamental problem that can only be mitigated through direct validation exercises. It is generally considered essential that sampling is performed at the start and preferably also the end of glider deployments to establish sensor performance and potentially identify effects of drift or bio-fouling. It is notable, however, that this study was successful in achieving a reasonable level of optical closure with independent ocean colour remote sensing data using manufacturer's calibrations for the triplet data. Monitoring the quality of match-up between remote sensing and glider-based estimates has potential to offer a new insight into sensor performance over the duration of a glider deployment.

This Barents Sea bio-optical model is based on a partial set of IOP data spanning the winter to summer time period, but with significant gaps and other limitations. For example, it was not possible to collect scattering and backscattering data on the summer cruise and *Chl* concentrations in winter were below the detection limit. As a result, the scattering and backscattering models are biased towards spring conditions, while the particulate absorption models are driven by spring and summer data. This potentially limits the applicability of the model and further work is needed to assess performance across different seasons and years. Only CDOM absorption was consistently measured across all three seasons and this was found to be relatively invariant. It should be noted that the relationship used to calculate *a*_CDOM_(440) from salinity (equation (2.7)) is limited to salinity greater than 27 PSU. This constraint did not impact our study because the gliders did not measure any values outside this range; however, there might be potential issues in areas with large freshwater influx due to melting ice or close to land. We suggest that a constant value of *a*_CDOM_(440) = 0.04 m^−1^ could be used in these water but, unfortunately, we did not have the data to test this approach. In addition, chlorophyll *a* concentration estimates from a fluorometer, as used for the development of bio-optical relationships in this study, are susceptible to bias introduced by choice of methodology. Estimates are also potentially affected by the presence of chlorophyll *b* in low concentration (data not shown). Nonetheless, the resulting bio-optical model produced reasonable estimates of absorption and backscattering profiles (figures 8 and 9) for both winter and spring (when optical profiles were available). For reasons that are not clear, modelling of attenuation profiles was less successful in winter (figure 8*b*) than in spring (figure 9*b*). This could be a consequence of a significant seasonal change in particulate optical properties, but this would be surprising, given the relative success of absorption and backscattering modelling across the same period. The optical clarity of the Barents Sea in winter when biological activity has been suppressed as a result of seasonal darkness is particularly challenging. It may be the case that the attenuation data in this case are less robust than elsewhere. Gaps in the *a*_nw_ and *c*_nw_ *in situ* profiles represent measurements where the iterative scattering correction [33] failed to converge. The disparity of modelled *c*_nw_ compared to *in situ* profiles can potentially be attributed to limited performance of the scattering correction in these clear waters, as well as a potential overestimation of *b_p_* by the bio-optical model when *Chl* concentration is low. Our findings show that the Barents Sea is a challenging environment optically and more work is needed to constrain bio-optical relationships, especially at times of low signal levels and constituent concentrations. While the bio-optical model presented here is unlikely to be an accurate description of relationships in other water bodies, the approach of developing the model and exploiting data collected by autonomous vehicles will be transferrable to other regions. The approach can almost certainly be applied to other types of autonomous platforms, such as bio-Argo floats. It should be noted, however, that some of these platforms are not suitable for deployments in shallow seas like the Barents Sea.

The development of the bio-optical model and subsequent validation was hindered by limited data availability, largely as a result of logistical constraints affecting participation in cruises and the amount of data that could be collected on busy multidisciplinary cruises with limited access to wire time. Difficult operating conditions, e.g. in the Arctic winter, also had an impact on the volume of data that could be collected. Most of these are common difficulties that regularly affect data collection at sea, with the challenge of operating in Arctic conditions exacerbating these problems. The approach we have taken has therefore necessarily involved making pragmatic decisions about how to best exploit the limited data available. Where possible, we have used existing datasets to inform our analysis and to provide broader context for the fitting of relationships. While the limited quantity of data available means that resulting bio-optical relationships must be viewed with an extra degree of caution (e.g. compared to extensive datasets used in other studies), the quality of the resulting match-up with ocean colour remote sensing data suggests that the bio-optical model provides a tolerable representation of the system and might be regarded as fit for purpose. Moreover, it is quite likely that operations involving glider deployments would involve generation of limited *in situ* data for calibration and validation, since part of the attraction of glider operations is to reduce time spent at sea. This study shows that careful exploitation of even quite limited datasets can provide useful outcomes.

## 5. Conclusion

The Arctic Ocean remains a systematically under-sampled ecosystem because its limited accessibility due to sea ice cover, prolonged periods of darkness and generally harsh operating conditions. Ocean colour remote sensing provides valuable synoptic views of the region, but all too frequently views are obscured by prevailing cloud and fog conditions that characterize the region. Low sun zenith angles and total darkness in winter months preclude data collection for a significant part of the year. Autonomous sampling platforms offer great potential to significantly expand observations in the region. The gliders used in this study were deployed from January to July, with a single turnover in spring, generating a unique seasonally resolved dataset that will offer new insight into the timing and development of algal production and relationships with physical oceanographic properties. Limited payload capacity and power consumption restrict the range of sensors that can be deployed on long duration missions such as this, and frequent recycling of gliders would defeat one of their key operational strengths. It is, therefore, imperative that we develop means to maximize exploitation of data generated by instruments such as the WET Labs optical triplet sensors that are regularly deployed on these platforms. Here, we have shown that even with quite limited sets of additional bio-optical observations, it is possible to construct bio-optical models that enable quantitative comparisons with satellite observations. The combination of *in situ* autonomous platforms and satellite remote sensing observations represents the popular perception of modern oceanographic sampling strategies. In reality, despite significant advances in both, there is relatively little material in the literature directly linking the two in this manner. The approach presented in this work is, we believe, both a useful demonstration of operational capability in itself, and has potential to be adapted to suit different operating conditions for other study sites. Future integration of glider and satellite observations will continue to require associated *in situ* sampling effort for general calibration /validation activities. Generation of regional and seasonal bio-optical models, along the lines presented here, is key to establishing a direct, quantitative link between autonomous *in situ* and satellite-based sampling technologies.

## Data accessibility

The article's supporting data can be accessed through BODC, the British Oceanographic Data Centre. *In situ* optical data: absorption and attenuation coefficients (winter http://dx.doi.org/10/dj87, spring http://dx.doi.org/10/dj89), backscattering coefficients (winter http://dx.doi.org/10/df6f, spring http://dx.doi.org/10/dgqd). Sample absorption (total, CDOM, particulate) data: CDOM (winter http://dx.doi.org/10/dj9h, spring http://dx.doi.org/10/dj9j, summer http://dx.doi.org/10/df6c), particulate (winter http://dx.doi.org/10/dkbz, spring http://dx.doi.org/10/dkb2, summer http://dx.doi.org/10/dkbq), total non-water (winter http://dx.doi.org/10/dkb4, spring http://dx.doi.org/10/dkb5, summer http://dx.doi.org/10/dkb3). Chlorophyll concentration data: spring (http://dx.doi.org/10/dg4m), summer (http://dx.doi.org/10/dg4b). Glider data: winter/spring (http://dx.doi.org/10/dmgm), spring/summer (http://dx.doi.org/10/dmkj).
